# Supplementary material for: Determinants of de novo B cell responses to drifted epitopes in post-vaccination SARS-CoV-2 infections
Source: medRxiv. 2023 Sep 14:2023.09.12.23295384. Preprint. [Version 2] doi: 10.1101/2023.09.12.23295384 (PMC10516057; doi:10.1101/2023.09.12.23295384)
Supplement: Supplement 1 [file NIHPP2023.09.12.23295384v2-supplement-1.pdf]

medRxiv preprint doi: https://doi.org/10.1101/2023.09.12.23295384; this version posted September 14, 2023. The copyright holder for this preprint (which was not certified by peer review) is the author/funder, who has granted medRxiv a license to display the preprint in perpetuity.
This article is a US Government work. It is not subject to copyright under 17 USC 105 and is also made available for use under a CC0 license.

Figure S1

A.

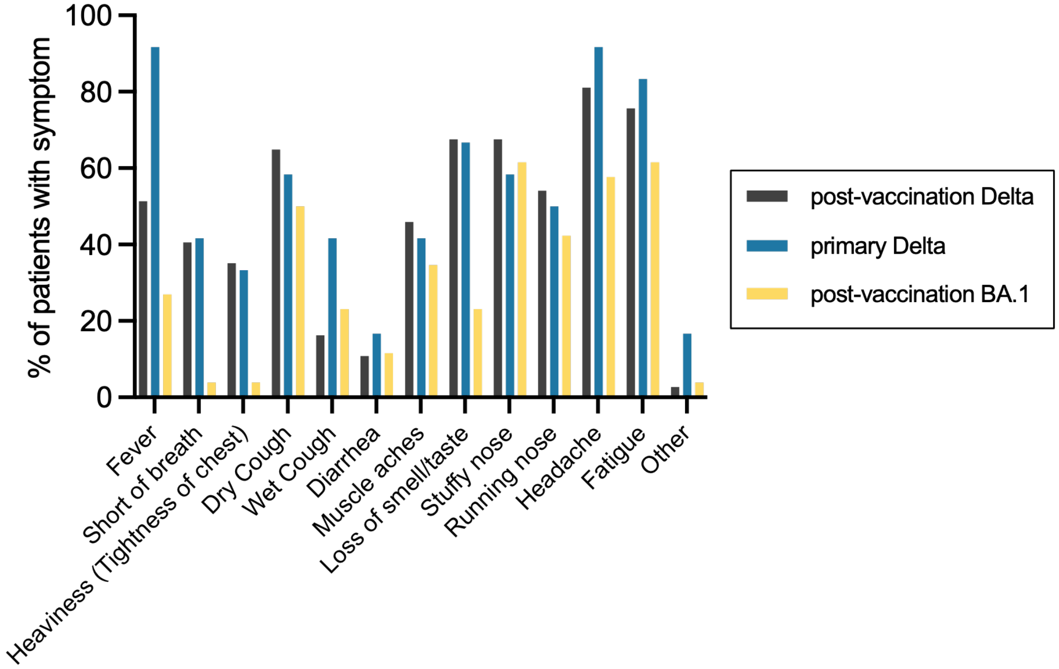

B.

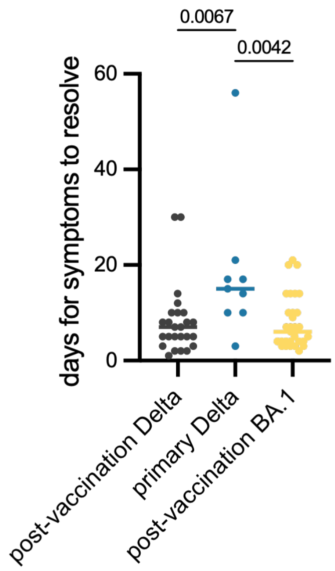

medRxiv preprint doi: https://doi.org/10.1101/2023.09.12.23295384; this version posted September 14, 2023. The copyright holder for this preprint (which was not certified by peer review) is the author/funder, who has granted medRxiv a license to display the preprint in perpetuity.
This article is a US Government work. It is not subject to copyright under 17 USC 105 and is also made available for use under a CC0 license.

Figure S1. Test All, Test Smart (TATS) symptom report. (A) Percentage of individuals from each TATS cohort that reported experiencing various respiratory/cold symptoms in study entry survey. **(B)** Reported days until symptoms resolved for each TATS cohort. Two-sided P values from t-test statistics were calculated for pairwise differences using one-way ANOVA. Post hoc testing for multiple comparisons between draws was performed using Tukey’s multiple comparisons test. P values greater than 0.05 are not depicted.

medRxiv preprint doi: https://doi.org/10.1101/2023.09.12.23295384; this version posted September 14, 2023. The copyright holder for this preprint (which was not certified by peer review) is the author/funder, who has granted medRxiv a license to display the preprint in perpetuity.
This article is a US Government work. It is not subject to copyright under 17 USC 105 and is also made available for use under a CC0 license.

Figure S2

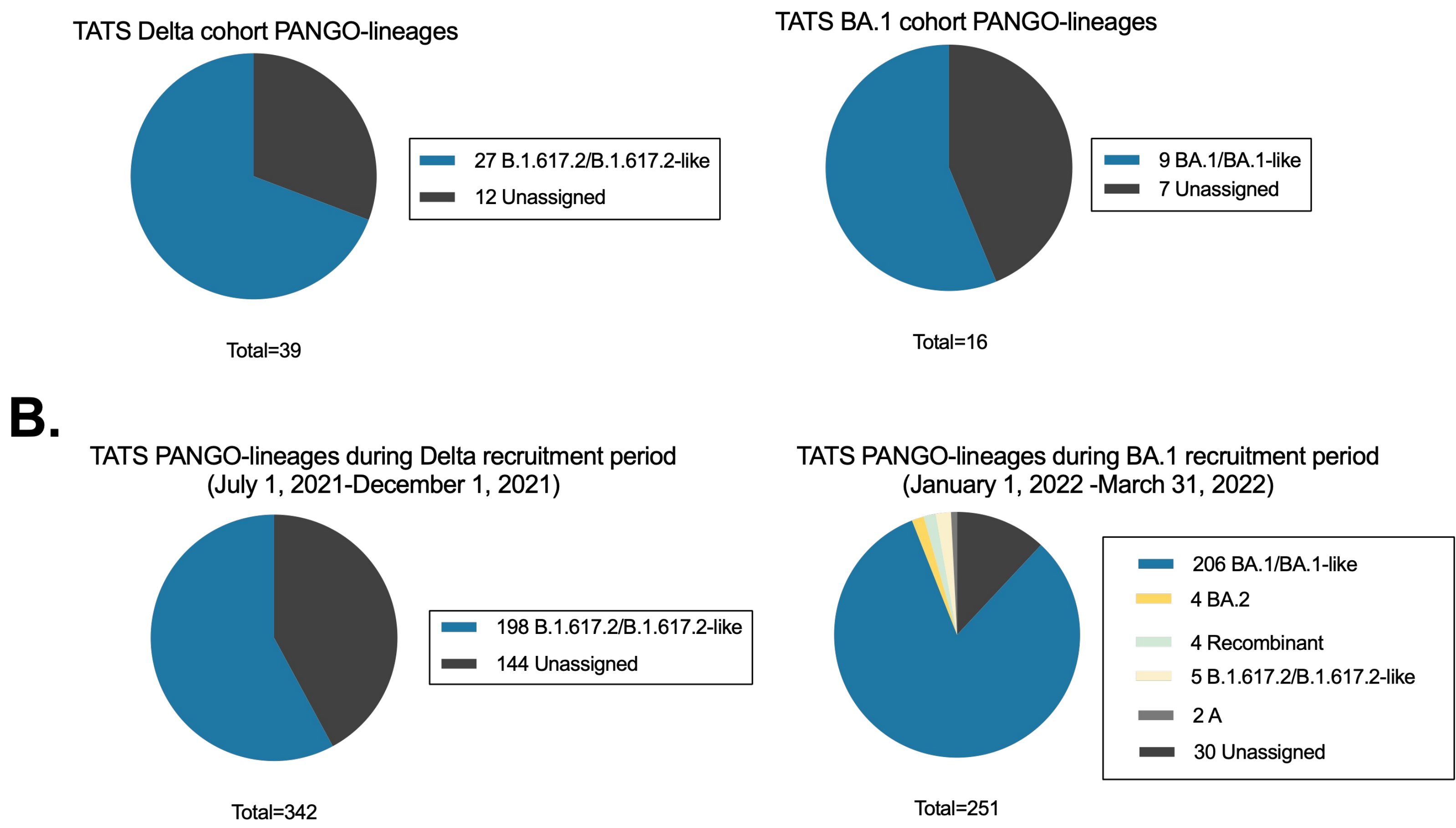

medRxiv preprint doi: https://doi.org/10.1101/2023.09.12.23295384; this version posted September 14, 2023. The copyright holder for this preprint (which was not certified by peer review) is the author/funder, who has granted medRxiv a license to display the preprint in perpetuity. This article is a US Government work. It is not subject to copyright under 17 USC 105 and is also made available for use under a CC0 license.

Figure S2. PANGO-lineage assignments from TATS PCR positive individuals. (A) Delta or BA.1 PANGO-lineage assignments after SARS-CoV-2 viral amplicon sequencing (Integrated DNA Technologies). Unassigned sequences could not be assigned to a PANGO-lineage due to insufficient viral RNA recovery and low sequence coverage. **(B)** PANGO-lineage assignments of all TATS samples submitted during the period of Delta cohort recruitment, July 1, 2021-December 1, 2021 (left panel) or during the period of BA.1 cohort recruitment, January 1, 2022-March 31, 2022 (right panel). Unassigned sequences could not be assigned a lineage due to insufficient viral RNA recovery and low sequence coverage.

medRxiv preprint doi: https://doi.org/10.1101/2023.09.12.23295384; this version posted September 14, 2023. The copyright holder for this preprint (which was not certified by peer review) is the author/funder, who has granted medRxiv a license to display the preprint in perpetuity. This article is a US Government work. It is not subject to copyright under 17 USC 105 and is also made available for use under a CC0 license.

Figure S3

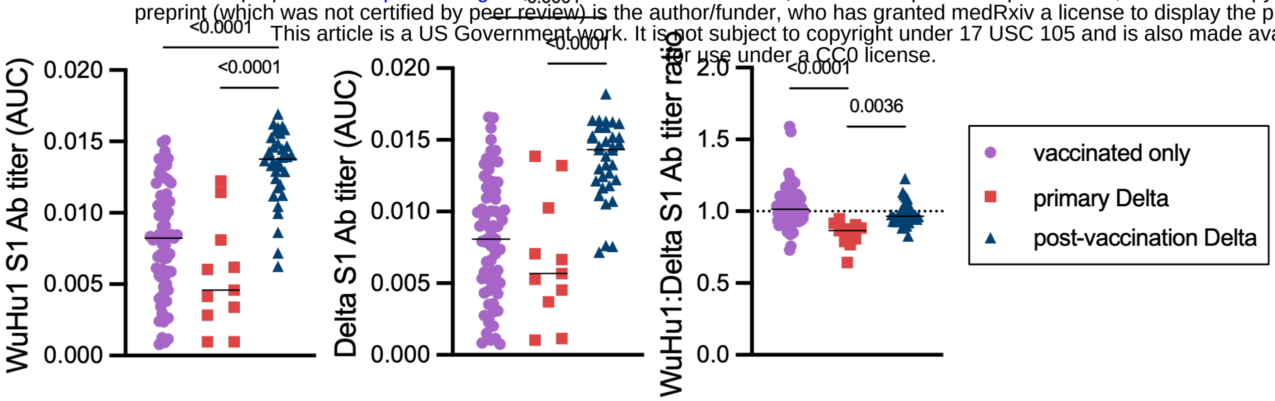

medRxiv preprint doi: https://doi.org/10.1101/2023.09.12.23295384; this version posted September 14, 2023. The copyright holder for this preprint (which was not certified by peer review) is the author/funder, who has granted medRxiv a license to display the preprint in perpetuity. This article is a US Government work. It is not subject to copyright under 17 USC 105 and is also made available for use under a CC0 license.

Figure S3. Primary and recall antibody responses to Wuhan and Delta strains of SARS-COV-2. Quantitative titers of WuHu1- and Delta S1-specific antibodies. Serum was initially diluted 1:60, serially diluted 1:3, assessed by ELISA for binding to the listed antigens, and area under the curve (AUC) values were calculated. Each symbol represents an individual. WuHu1 AUC values were divided by their Delta AUC titer in the same individual to calculate a WuHu1:Delta S1 ratio in the rightmost panel. Two-sided P values from t-test statistics were calculated for pairwise differences using one-way ANOVA. Post hoc testing for multiple comparisons between draws was performed using Tukey's multiple comparisons test. P values greater than 0.05 are not depicted.

medRxiv preprint doi: https://doi.org/10.1101/2023.09.12.23295384; this version posted September 14, 2023. The copyright holder for this preprint (which was not certified by peer review) is the author/funder, who has granted medRxiv a license to display the preprint in perpetuity. This article is a US Government work. It is not subject to copyright under 17 USC 105 and is also made available for use under a CC0 license.

Figure S4

primary Delta

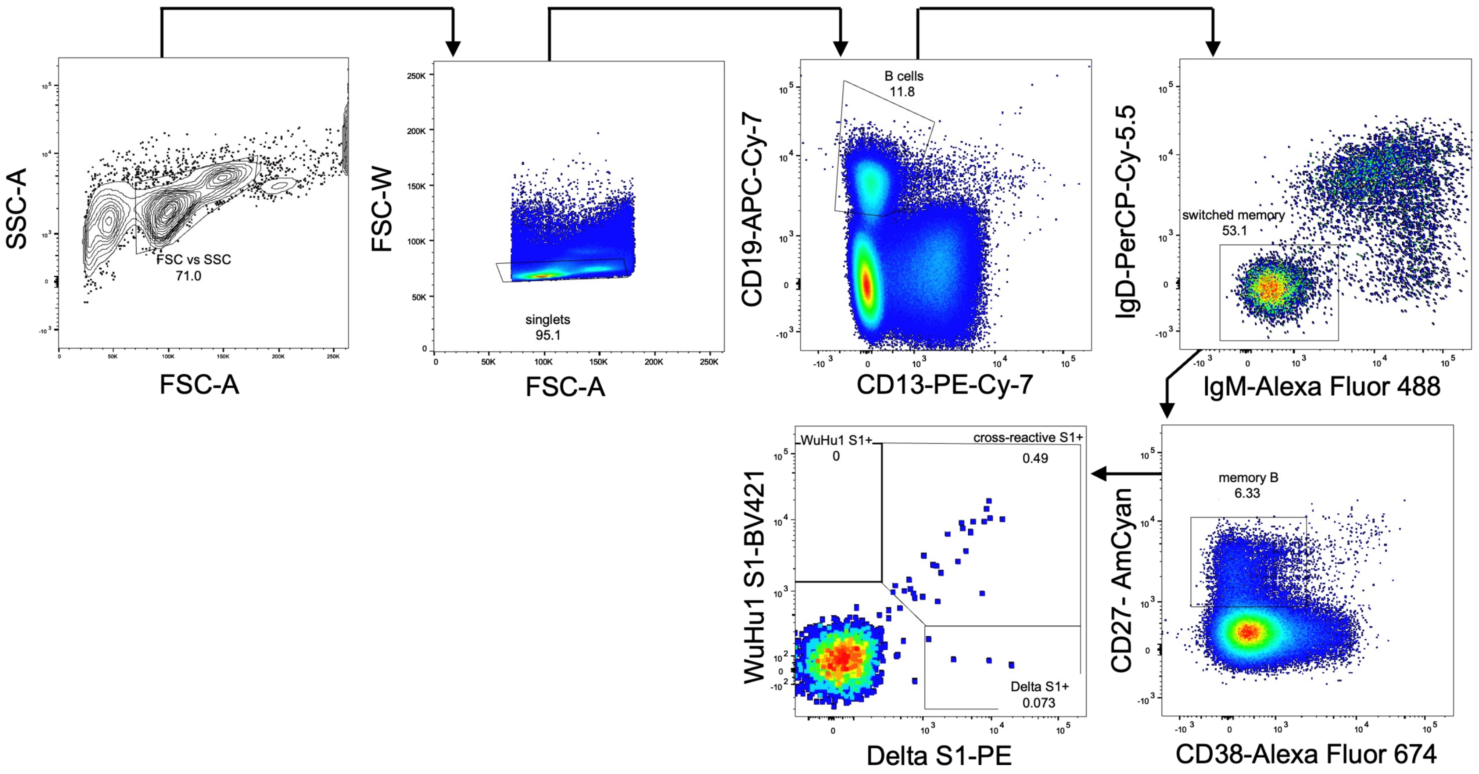

post-vaccination Delta

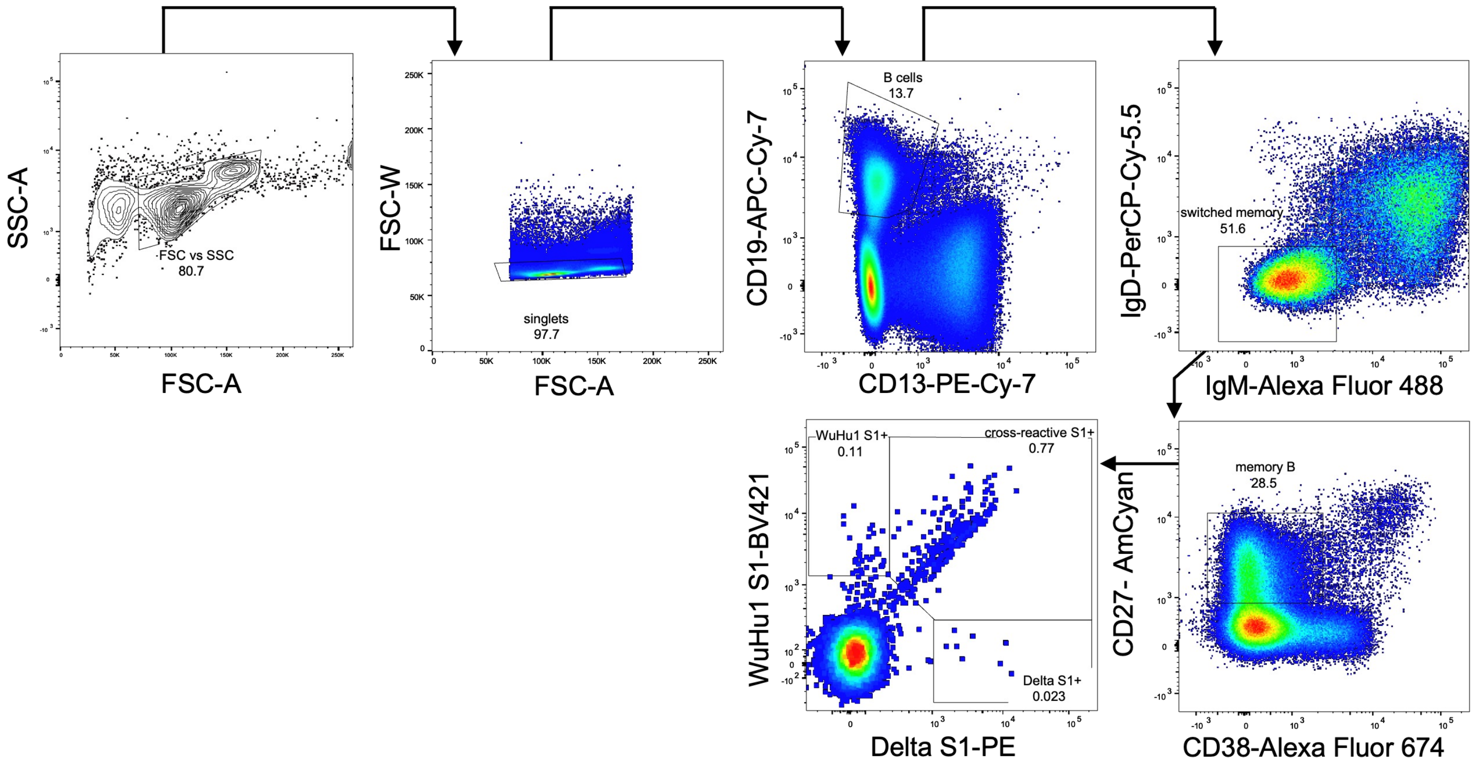

medRxiv preprint doi: https://doi.org/10.1101/2023.09.12.23295384; this version posted September 14, 2023. The copyright holder for this preprint (which was not certified by peer review) is the author/funder, who has granted medRxiv a license to display the preprint in perpetuity.
This article is a US Government work. It is not subject to copyright under 17 USC 105 and is also made available for use under a CC0 license.

Figure S4. Flow cytometric gating strategy with Delta S1 and WuHu1 S1 tetramers. Examples of a sample from a primary Delta infection (top) and post-vaccination Delta infection (bottom) are shown.

medRxiv preprint doi: https://doi.org/10.1101/2023.09.12.23295384; this version posted September 14, 2023. The copyright holder for this preprint (which was not certified by peer review) is the author/funder, who has granted medRxiv a license to display the preprint in perpetuity.
This article is a US Government work. It is not subject to copyright under 17 USC 105 and is also made available for use under a CC0 license.

Figure S5

A.

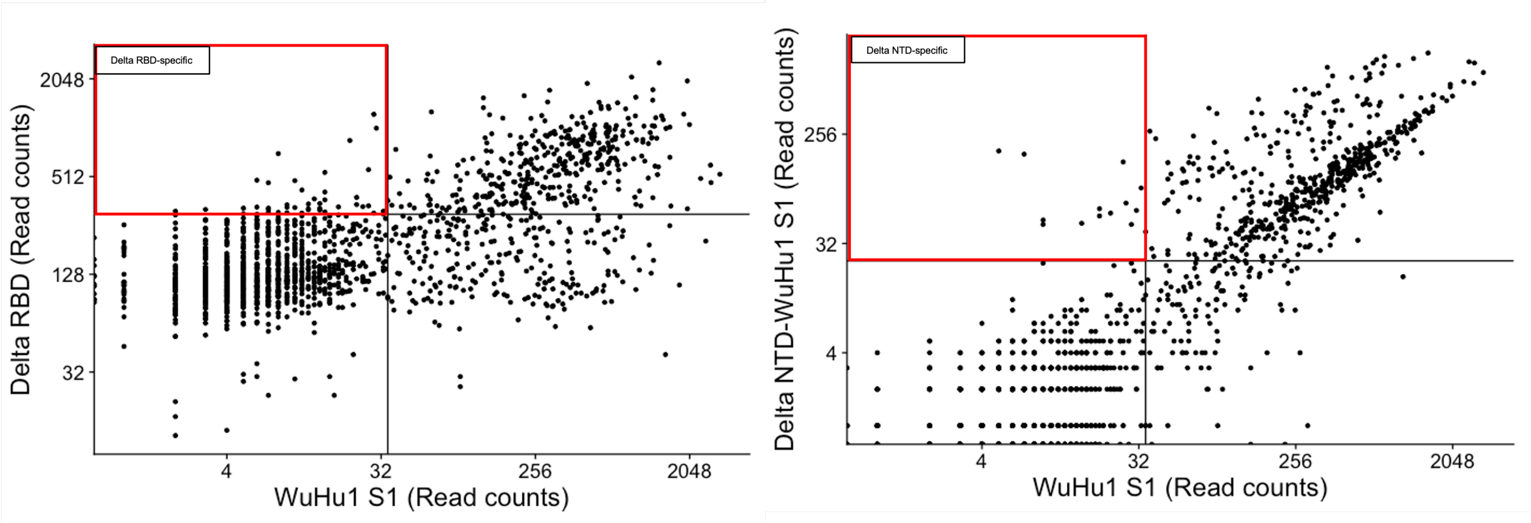

B.

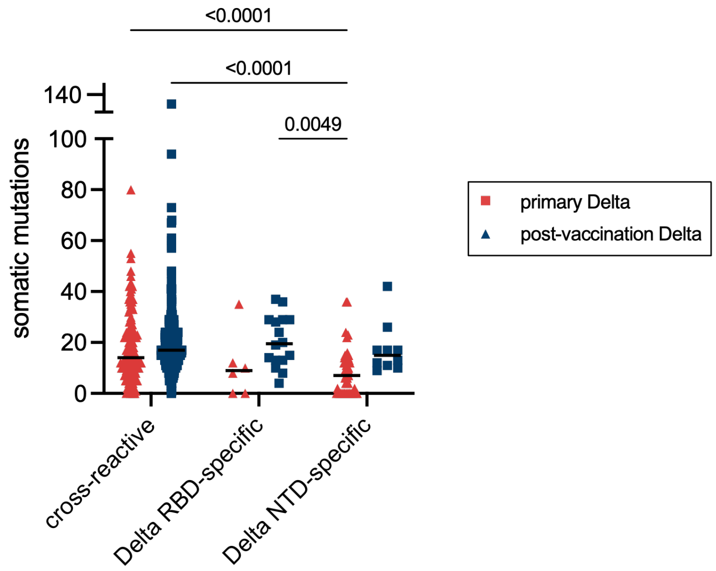

C.

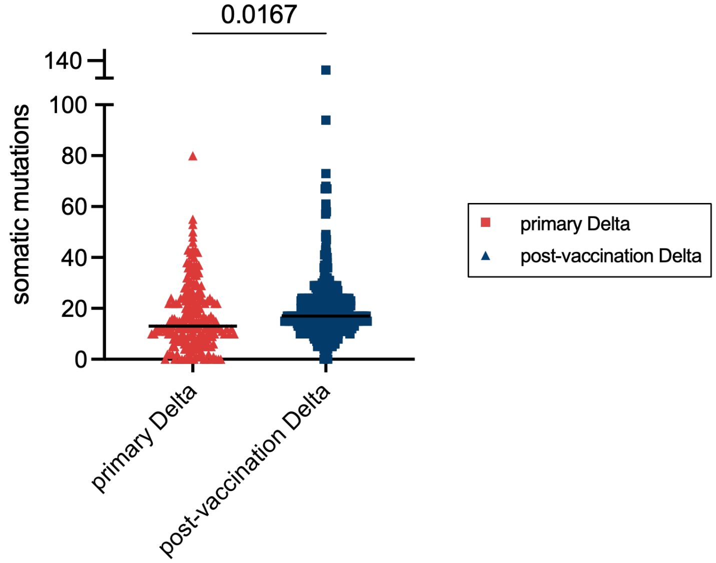

medRxiv preprint doi: https://doi.org/10.1101/2023.09.12.23295384; this version posted September 14, 2023. The copyright holder for this preprint (which was not certified by peer review) is the author/funder, who has granted medRxiv a license to display the preprint in perpetuity. This article is a US Government work. It is not subject to copyright under 17 USC 105 and is also made available for use under a CC0 license.

Figures S5. LIBRA-seq analysis in primary and post-vaccination Delta infections and quantification of somatic mutations. (A) A chimeric protein (Delta NTD-WuHu1 S1) was generated in which Delta NTD mutated epitopes (T19R, G142D, E156-, F157-, R158G) were incorporated into the otherwise WuHu1 S1 backbone. Quantification of Delta RBD-specific **(left)** and Delta NTD-specific memory B cells **(right)** in individuals that experienced a post-vaccination Delta infection. Delta RBD-specific cells were classified by cells that had Delta RBD read counts of greater than 300 and WuHu1 S1 read counts of less than 35. Delta NTD-specific cells were classified by cells that had Delta NTD-WuHu1 S1 read counts of greater than 23 and WuHu1 S1 read counts of less than 35. Read count thresholds to determine positivity were set using samples in which cells lacking Spike-binding specificities were sorted and sequenced. Plots are concatenated from ten individuals. **(B)** Somatic mutations were calculated using the observedMutations command in the Shazam Immcantation package in R. Specificities of cells are determined using the same cutoffs described in **Figure S3A and 3D. (C)** Quantification of somatic mutations of all Spike specific cells subjected to scRNAseq from either ten primary or post-vaccination Delta infections.

medRxiv preprint doi: https://doi.org/10.1101/2023.09.12.23295384; this version posted September 14, 2023. The copyright holder for this preprint (which was not certified by peer review) is the author/funder, who has granted medRxiv a license to display the preprint in perpetuity.
This article is a US Government work. It is not subject to copyright under 17 USC 105 and is also made available for use under a CC0 license.

Figure S6

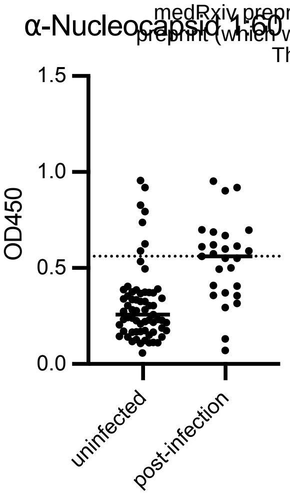

medRxiv preprint doi: https://doi.org/10.1101/2023.09.12.23295384; this version posted September 14, 2023. The copyright holder for this preprint (which was not certified by peer review) is the author/funder, who has granted medRxiv a license to display the preprint in perpetuity. This article is a US Government work. It is not subject to copyright under 17 USC 105 and is also made available for use under a CC0 license.

Figure S6. anti-Nucleocapsid titers in uninfected individuals. Individuals with α-Nucleocapsid titers of greater than 0.6 at a1:60 serum dilution were considered previously infected and excluded from the study.

medRxiv preprint doi: https://doi.org/10.1101/2023.09.12.23295384; this version posted September 14, 2023. The copyright holder for this preprint (which was not certified by peer review) is the author/funder, who has granted medRxiv a license to display the preprint in perpetuity. This article is a US Government work. It is not subject to copyright under 17 USC 105 and is also made available for use under a CC0 license.

Figure S7

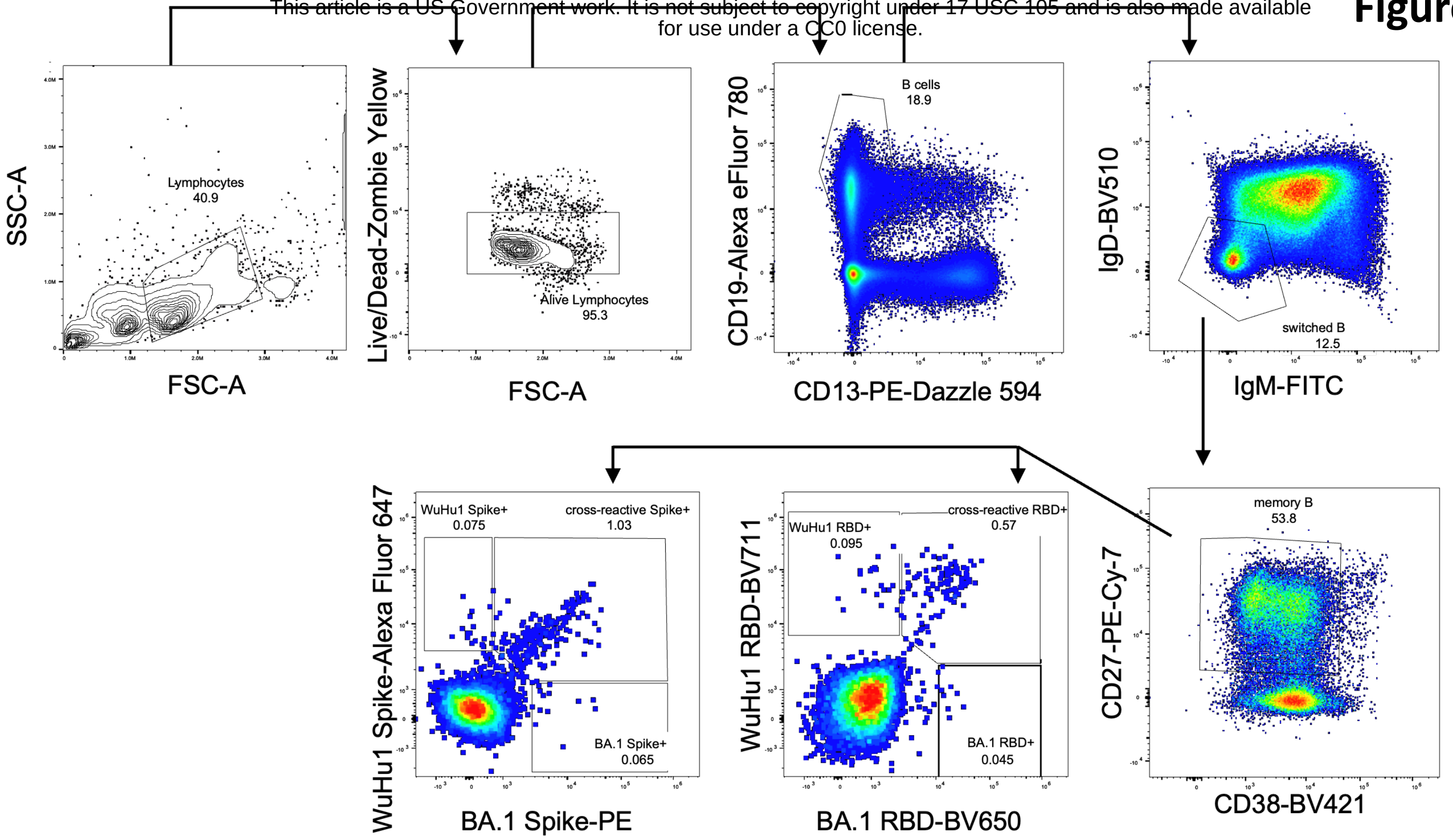

medRxiv preprint doi: https://doi.org/10.1101/2023.09.12.23295384; this version posted September 14, 2023. The copyright holder for this preprint (which was not certified by peer review) is the author/funder, who has granted medRxiv a license to display the preprint in perpetuity. This article is a US Government work. It is not subject to copyright under 17 USC 105 and is also made available for use under a CC0 license.

Figure S7. Flow cytometric gating strategy with BA.1 RBD, BA.1 Spike, WuHu1 RBD and WuHu1 Spike tetramers. An example of a sample from a post-vaccination BA.1 infection is shown.

medRxiv preprint doi: https://doi.org/10.1101/2023.09.12.23295384; this version posted September 14, 2023. The copyright holder for this preprint (which was not certified by peer review) is the author/funder, who has granted medRxiv a license to display the preprint in perpetuity.
This article is a US Government work. It is not subject to copyright under 17 USC 105 and is also made available for use under a CC0 license.

Figure S8

A.

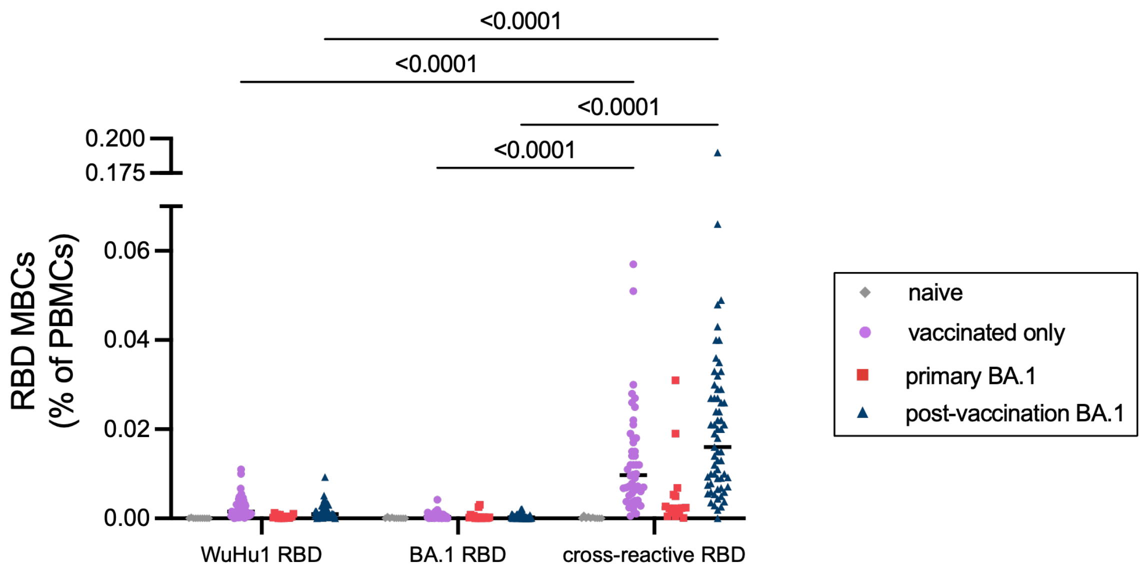

B.

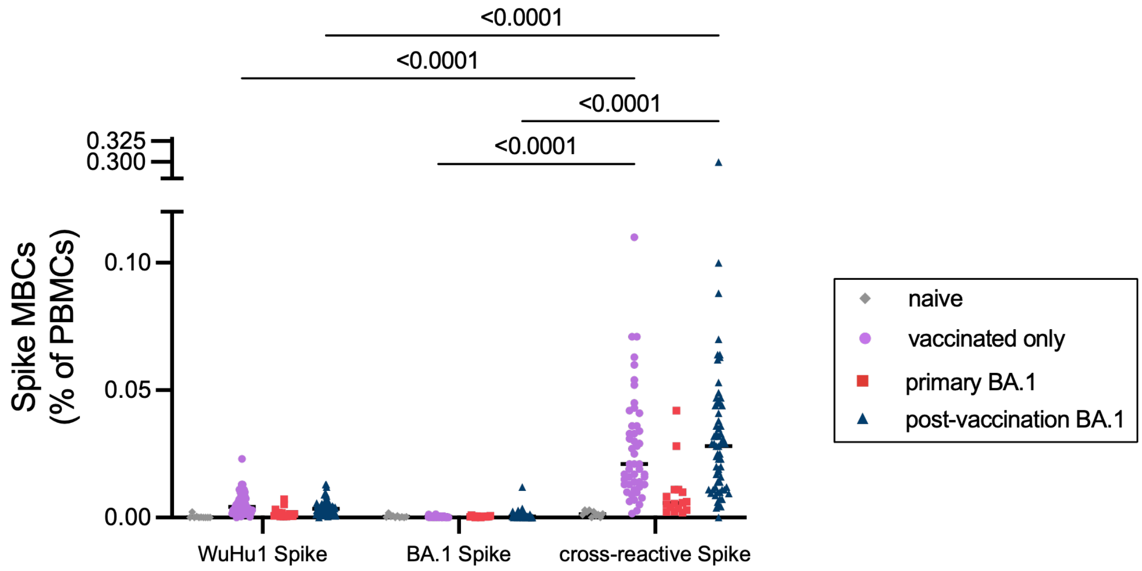

medRxiv preprint doi: https://doi.org/10.1101/2023.09.12.23295384; this version posted September 14, 2023. The copyright holder for this preprint (which was not certified by peer review) is the author/funder, who has granted medRxiv a license to display the preprint in perpetuity. This article is a US Government work. It is not subject to copyright under 17 USC 105 and is also made available for use under a CC0 license.

Figure S8. WuHu1 and BA.1 Memory B cell flow cytometric quantification. (A) Cells that bind both WuHu1 RBD and BA.1 RBD are annotated as cross-reactive RBD+, whereas cells that bind only WuHu1 RBD or BA.1 RBD are annotated as WuHu1 RBD+ or BA.1 RBD+, respectively. Quantification of isotype-switched memory B cells as a percentage of total PBMCs for Wuhu1 RBD+, BA.1 RBD+ and cross-reactive RBD+ specificities for each cohort of SARS-CoV-2 immune histories. Each symbol represents an individual. Two-sided P values from t-test statistics were calculated for pairwise differences using two-way ANOVA. Post hoc testing for multiple comparisons between draws was performed using Tukey's multiple comparisons test. P values greater than 0.05 are not depicted. **(B)** Cells that bind both WuHu1 RBD and BA.1 Spike are annotated as cross-reactive Spike+, whereas cells that bind only WuHu1 Spike or BA.1 Spike are annotated as WuHu1 Spike+ or BA.1 Spike+, respectively. Quantification of isotype-switched memory B cells for Wuhu1 Spike+, BA.1 Spike+ and cross-reactive Spike+ specificities for each cohort of SARS-CoV-2 immune histories. Each symbol represents an individual. Two-sided P values from t-test statistics were calculated for pairwise differences using two-way ANOVA. Post hoc testing for multiple comparisons between draws was performed using Tukey's multiple comparisons test. P values greater than 0.05 are not depicted.
